# Efficacy and safety of tenofovir alafenamide versus tenofovir disoproxil in chronic hepatitis b: a systematic review and meta-analysis of randomized controlled trials

**DOI:** 10.1186/s12879-026-13974-4

**Published:** 2026-07-06

**Authors:** Marrium Sultan Dar, Haziq Ovais, Aimen Waqar Khan, Samra Ishaq, Yusra Muhammad Saleem, Syeda Dua Azhar, Muhammad Affan, Zuhaa Rehman, Fabiha Athar, Tooba Fatima, Jawad Zahid, Saqib Ali, Noor ul Ain, Salim Surani, Muhammad Sohaib Asghar, Khabab Abbasher Hussien Mohamed Ahmed

**Affiliations:** 1 Department of Medicine Institute, Jinnah Sindh Medical University, Karachi, Pakistan; 2 Department of Medicine Institute, Texas A&M university, Austin, TX USA; 3 Department of Medicine, Mayoclinic, Rochester, MN USA; 4 Faculty of Medicine Institute, University of Khartoum, Khartoum, 11111 Sudan; 5 Department of Pathophysiology, School of Medicine, St. George’s University, True Blue, St. George’s, West Indies, Grenada

**Keywords:** Hepatitis B, Tenofovir, Chronic hepatitis B, Viral load, Meta-analysis

## Abstract

**Background:**

In treating Chronic Hepatitis B (CHB), tenofovir disoproxil fumarate (TDF) carries risks like renal toxicity and reduced bone mineral density (BMD). TAF, the next-generation prodrug of TFV, is more stable in plasma than TDF, requires a significantly lower dose (25 mg vs. 300 mg), and results in approximately 90% lower plasma TFV levels, contributing to a more favorable renal and bone safety profile. This study aimed to evaluate the efficacy and safety of tenofovir alafenamide fumarate (TAF) compared to TDF in CHB.

**Methods:**

We conducted a search on PubMed, Google Scholar, and the Cochrane Library. Only four RCTs that studied the comparison of efficacy and safety of TAF and TDF in treating CHB were included. The Primary outcome of interest was the proportion of patients with Hepatitis B virus (HBV) DNA below 15–29 IU/ml as all the RCT’s included in this study report the same HBV DNA levels. We used a random-effects model to calculate the Risk Ratio (RR) and Mean Difference (MD) with 95% CI.

**Results:**

This meta-analysis includes four eligible RCTs including 1,960 total patients. The comparison between TAF and TDF groups regarding HBV DNA level revealed no significant difference (RR = 1.00, 95% CI = 0.96 to 1.05; *P* = 0.82). Pooled data showed beneficial effects of TAF compared with TDF for ALT Normalization (RR = 1.38; 95% CI = 1.16 TO 1.64; *p* = 0.0002), Hip BMD (MD = 1.44, 95% CI = 0.98 to 1.91; P ‹ 0.00001), Spine BMD (MD = 1.93, 95% CI = 1.42 to 2.44; P ‹ 0.00001), serum creatinine concentration (MD= -0.02, 95% CI=-0.03 to -0.01; P ‹ 0.00001) and eGFR (MD = 3.55, 95% CI = 2.97 to 4.14; P ‹ 0.00001). However, the TAF group showed a significantly greater proportion of patients (46/1080 vs. 5/662) with LDL levels > 190 or 300 mg/dl than the TDF group (RR = 4.95, 95% CI = 1.21 to 20.29; *P* = 0.03).

**Conclusion:**

The TAF demonstrated improved BMD and renal function compared to TDF. There was no significant difference between TAF and TDF in achieving viral suppression.

**Clinical trial number:**

This was a systematic review and meta-analysis not a clinical trial.

**Study registration:**

This systematic review and meta-analysis is registered at PROSPERO (CRD42023456006).

**Supplementary Information:**

The online version contains supplementary material available at https://doi.org/10.1186/s12879-026-13974-4.

## Introduction

Over 250 million people live with chronic hepatitis B (CHB), a leading cause of liver cancer and cirrhosis [1]. According to a 2019 World Health Organization (WHO) estimate, approximately 296 million people are living with CHB globally [2]. Hepatitis B virus (HBV) is a partially double-stranded DNA virus with a core antigen (HBcAg) surrounded by a shell containing a surface antigen (HBsAg). Chronic HBV infection is defined by the persistence of HBsAg for more than six months [3].

HBV transmission occurs through two primary routes: vertical transmission from mother to newborn at birth, and horizontal transmission through contaminated blood or body fluids, as well as high-risk practices such as injection drug use, tattooing, and acupuncture [3, 4]. Without treatment, chronic HBV leads to progressive hepatic fibrosis, cirrhosis, liver decompensation, and, most critically, hepatocellular carcinoma (HCC) [5].

Current treatment options can improve patient outcomes; however, HBV cannot be permanently eradicated, leaving individuals at a baseline risk of viral reactivation [6]. Nearly all patients experience viral relapse upon treatment discontinuation, making lifelong therapy necessary [6]. The ideal antiviral agent for CHB should therefore combine a high barrier to resistance, a favorable safety profile, and sustained efficacy across all patient groups [7].

Current HBV treatment encompasses two broad drug classes: pegylated interferon-alpha (PEG-IFN-α), and direct-acting antivirals (DAAs) including nucleotide analogues — adefovir dipivoxil (ADV), tenofovir disoproxil fumarate (TDF), and tenofovir alafenamide fumarate (TAF) — and nucleoside analogues such as lamivudine (LMV), telbivudine (LdT), and entecavir (ETV) [8, 9]. Major international clinical practice guidelines from the American Association for the Study of Liver Diseases (AASLD), the European Association for the Study of the Liver (EASL), and the Asian-Pacific Association for the Study of the Liver (APASL) recommend nucleos(t)ide analogues, particularly entecavir and tenofovir, as preferred first-line therapy for chronic hepatitis B [19–23]. TDF, the established first-line treatment for CHB, is an orally bioavailable prodrug of tenofovir, a nucleotide analogue reverse transcriptase inhibitor [7]. Following oral administration, TDF is cleaved to release tenofovir (TFV), which is absorbed into the systemic circulation and taken up by hepatocytes, where it undergoes phosphorylation to tenofovir diphosphate (TFV-DP). TFV-DP potently inhibits HBV polymerase in a competitive manner, suppressing viral replication and reducing HBV DNA levels [10].

Despite its efficacy, long-term TDF use has been associated with clinically meaningful declines in renal function and bone mineral density (BMD), as evidenced by data from trials with up to five years of follow-up [11]. These safety signals prompted the development of a next-generation alternative.

In response to these concerns, tenofovir alafenamide fumarate (TAF), a next-generation oral prodrug of TFV, was developed [12]. Like TDF, TAF is a potent nucleotide reverse transcriptase inhibitor; however, it is activated intracellularly — converting to TFV within the target hepatocyte — rather than in the systemic circulation. TAF is more stable in plasma than TDF, enabling effective viral suppression at a substantially lower dose (25 mg vs. 300 mg). This results in plasma TFV levels approximately 90% lower and intracellular TFV-diphosphate (TFV-DP) concentrations in peripheral blood mononuclear cells (PBMCs) more than five-fold higher compared with TDF [14, 15]. In long-term follow-up of two double-blind randomized controlled trials, Chan et al. reported that TAF maintained high rates of viral suppression with a more favorable renal and bone safety profile than TDF [13].

Given the distinct pharmacological profiles and safety considerations of TAF and TDF, a rigorous head-to-head comparison is warranted to inform clinical decision-making. This systematic review and meta-analysis aims to compare the efficacy and safety of TAF versus TDF in patients with chronic hepatitis B.

## Materials and methods

### Search strategy

According to the 2020 PRISMA (Preferred Reporting Items for Systematic Review and Meta-Analysis) guidelines, we conducted a methodical literature search from the inception till August 8, 2023, on PubMed, Google Scholar, and Cochrane Library.

**Fig. 1:**
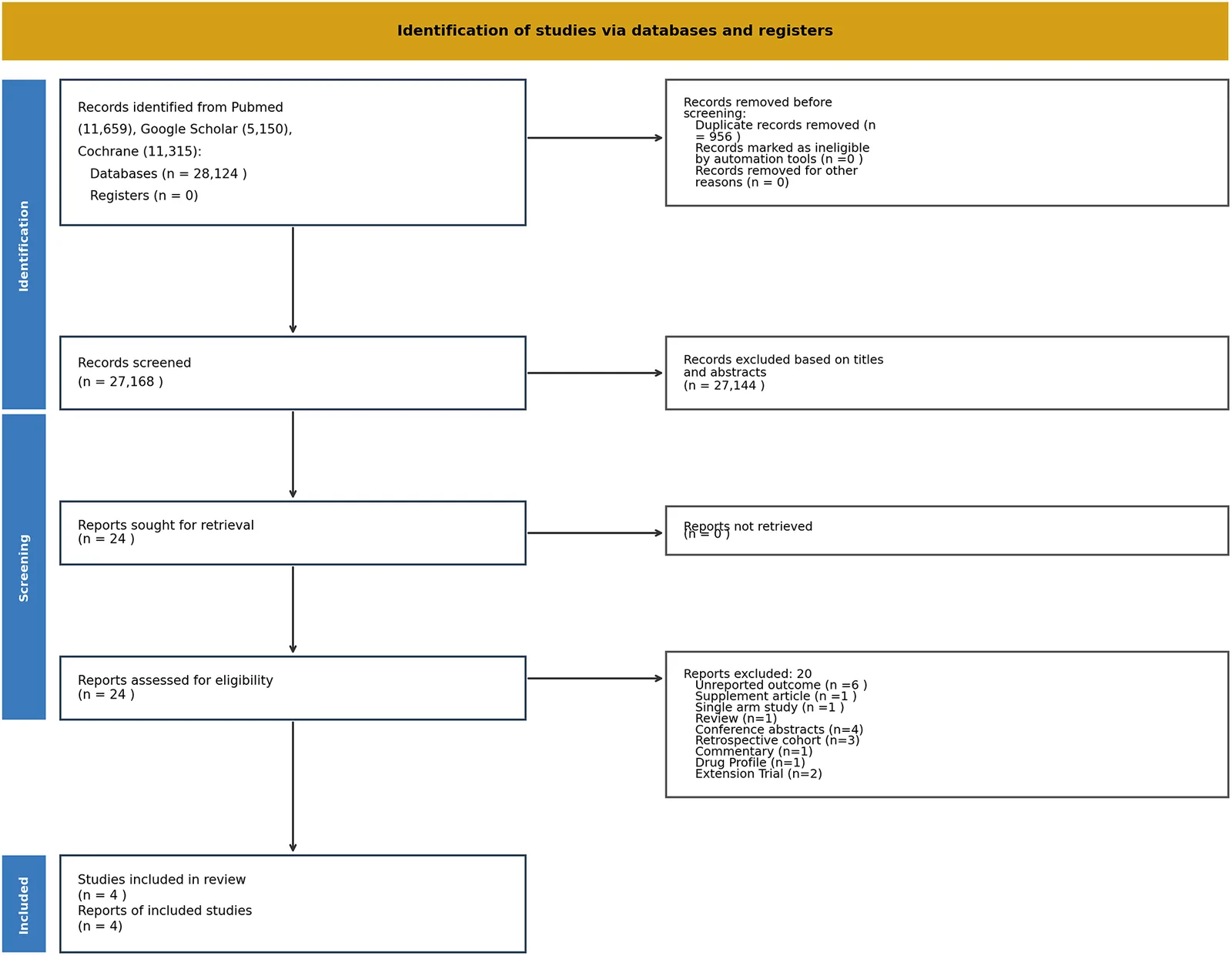
PRISMA flowchart of search results

A comprehensive search strategy was made using Mesh terms and boolean operators and is given in Supplementary Table S1. Two authors (MA and SA) reviewed the titles, abstracts, full texts, and reference lists of all identified studies, and extracted and cross checked the articles. Authors resolved any disagreements by discussion. Reference lists from related primary studies and review articles were also scrutinized for additional relevant studies. A PRISMA flowchart describing the literature selection is presented in Fig. 1.

### Eligibility criteria

#### Inclusion criteria

Based on the PICO approach (P = CHB patients; I = Tenofovir Alafenamide; C = Tenofovir Disoproxil; O = explained later), we narrowed inclusion criteria for the studies according to outcomes and study type when they (1) (a) evaluated the efficacy of TAF versus TDF in CHB (HBV DNA levels and Alanine transaminase (ALT) Normalization) and (b) reported the safety profile in terms of mean percentage change in hip and spine BMD, serum creatinine, estimated glomerular filtration rate (eGFR), and low-density lipoprotein (LDL) cholesterol (2) are a randomized controlled trial (RCT) in English with full text accessible for review. There was no restriction regarding country, race, and the year of publication but only full text English language articles were added. To preserve causal inference, we restricted our meta-analysis to Randomized Controlled Trials (RCTs). Pooling non-randomized studies introduces severe confounding and selection bias, a known methodological pitfall that artificially inflates effect sizes and invalidates the validity of pooled treatment or vaccine efficacy estimates.

Full-text articles and published abstracts of relevant RCTs were included. In addition, reference lists of selected papers were manually searched to identify further relevant articles.

#### Exclusion criteria

Exclusion criteria are as follows: (1) case reports, case series, conference abstracts, and observational studies; (2) having more than two interventions assessed; (3) studies having missing data.

No articles were excluded based on quality, sample size, or other objective criteria relevant to the study design and analysis.

### Data extraction

For articles published from inception to date, each reviewer extracted data onto a standardized, pre-piloted extraction form independently, without access to the other reviewer’s entries. Following independent extraction, both forms were compared side-by-side, and any discrepancies in numerical values, outcome definitions, or study characteristics were flagged and reconciled by returning to the full-text article. Two reviewers (MA and SA) screened all identified trials based on title, abstract, and full text, and any doubt was discussed with another independent reviewer (MSD) to resolve any differences. Final inclusion was based on consensus between the three reviewers. We extracted the main characteristics of each included study: (1) publication details (author’s name, year of publication, study design, sample size, and study duration); (2) baseline characteristics of the studied population (age, gender, intervention indication, follow-up duration)(3) Primary outcome - proportion of patients with HBV DNA below 15–29 IU/ml (Buti et al. and Chan et al. used < 29 IU/ml as the cutoff, Lampertico et al. used < 20 IU/ml as the cut-off while Byun et al. used < 15 IU/ml as the cut-off); (4) secondary outcomes - ALT Normalization (by AASLD), Hip and Spine BMD, serum creatinine concentration, eGFR and proportion of patients with LDL cholesterol above 190–300 mg/dl. The extracted data were entered into an MS Excel spreadsheet for further analysis. The authors double-checked all entries before performing analysis. BMD at the hip and spine was assessed by dual-energy X-ray absorptiometry at week 48 in all four included trials. Sample sizes available for these outcomes ranged from 174 to 873 participants per trial: Buti et al. [7] (*n* = 425; 285 TAF vs. 140 TDF), Chan et al. [16] (*n* = 873; 581 TAF vs. 292 TDF), Lampertico et al. [17] (*n* = 488; 243 TAF vs. 245 TDF), and Byun et al. [18] (*n* = 174; 87 TAF vs. 87 TDF).

### Risk of bias assessment

We assessed the quality and risk of bias of the RCTs included in this study with the modified Cochrane Collaboration’s risk of bias tool (Rob 1 version 6.4). Two studies had a high risk of bias due to incomplete outcome reporting and open-label design. Results from the risk of bias assessment were graphed (Fig. S1), and the potential problems or limitations were highlighted. Each included RCT was evaluated across seven domains using the Cochrane Risk of Bias tool (RoB 1.0): (1) random sequence generation (selection bias); (2) allocation concealment (selection bias); (3) blinding of participants and personnel (performance bias); (4) blinding of outcome assessment (detection bias); (5) incomplete outcome data (attrition bias); (6) selective reporting (reporting bias); and (7) other bias. As illustrated in Fig. S1, the majority of included studies demonstrated low risk of bias across most domains. Random sequence generation and incomplete outcome data (attrition bias) were rated low risk in nearly all studies. However, allocation concealment and blinding of participants and personnel (performance bias) showed high risk of bias in approximately 25% of studies, likely attributable to the open-label design of several trials. Blinding of outcome assessment (detection bias) showed the greatest concern, with approximately 25% of studies rated as unclear risk and an additional 25% rated as high risk. Selective reporting (reporting bias) was rated high risk in approximately 25% of studies. Other bias was rated low risk across all included trials.

### Statistical analysis

We used Cochrane Collaboration’s software Review Manager (RevMan version 5.4.1) to perform the meta-analysis. One of the authors conducted Data analysis of the primary and secondary outcomes of interest using the random effects model (DerSimonian and Laird inverse variance). The Risk Ratio (RR) of the primary outcome, effect on HBV DNA, and secondary outcome, ALT Normalization, were pooled using a inverse variance with 95% confidence intervals (CI). A pooled effect of mean differences (MD) was calculated using the remaining secondary continuous outcome functions with the given mean values and standard deviations (SDs). Heterogeneity between studies was evaluated using Higgins I^2^ statistics. Heterogeneity > 50% was considered significant. The forest plots were generated to assess the pooled data. The meta-analysis results were reported as pooled effects, with corresponding 95% CIs and p-values less than 0.05 were considered significant in all cases.

## Results

### Study selection

Of the initial 28,124 articles generated from the database search, 956 were eliminated due to duplication. Subsequently, 27,168 records underwent screening based on their initial identification, excluding 27,144 records due to inadequate relevance found in their titles and abstracts. The large initial yield was due to broad search terms including HIV studies, which were later excluded. Following this screening phase, 24 reports were identified for retrieval. Of these, only four reports met the criteria based on full text and were included in the final meta-analysis, and all were RCT.

### Baseline characteristics

The Baseline characteristics of the included RCTs are shown in Tables 1 and 2. Four eligible RCTs were included in the present meta-analysis, including 1,960 patients. The publication years of the involved articles varied from 2016 to 2022. In the meta-analysis, all four RCTs included in the study utilized interventions involving TAF as the treatment group and TDF as the comparator group [7, 16–18]. The efficacy endpoints in all the RCTs were assessed at 48 weeks. The detailed characteristics of included RCTs are presented in Table S2.

**Table 1:** Baseline characteristics of included studies

Study	Study Design	Total no. of patients	No. of patients		Age Mean ± SD¹		HBeAg Status
			Control Group	Intervention Group	Control Group	Intervention Group	
Buti 2016 [7]	RCT	425	140	285	48 ± 10	45 ± 12	HBeAg negative
Chan 2016 [16]	RCT	873	292	581	38 ± 12	38 ± 11	HBeAg positive
Lampertico 2020 [17]	RCT	488	245	243	51 ± 10.8	51 ± 10.5	Both HBeAg negative and positive
Byun 2022 [18]	RCT	174	87	87	53.3 ± 9.5	56.4 ± 9.4	Both HBeAg negative and positive

^1^SD: Standard Deviation

**Table 2:** Baseline HBV DNA, ALT, Hip BMD, Spine BMD, Serum Creatinine, eGFR¹HBV: Hepatitis B Virus; ²ALT: Alanine transaminase; ³ BMD: Bone Mineral Density; ⁴eGFR: Estimated Glomerular Filtration Rate

Study	HBV DNA¹ Below 15–29 log_10_ IU/ml *N*(%)		ALT² Normalization *N*(%)		Hip BMD³ Mean ± SD OR *N*(%)		Spine BMD Mean ± SD OR *N*(%)		Serum Creatinine Mean ± SD/IQR		eGFR⁴Mean ± SD	
	Control Group	Intervention Group	Control Group	Intervention Group	Control Group	Intervention Group	Control Group	Intervention Group	Control Group	Intervention Group	Control Group	Intervention Group
Buti 2016 [7]	4(3)	13(5)	121(86)	236(83)	0.94 ± 0.14	0.95 ± 0.16	1.08 ± 0.18	1.05 ± 0.19			100.3 ± 24.2	104.7 ± 27.8
Chan 2016 [16]	7.6(1.41)	7.6(1.34)	268(92)	537(92)	0.96 ± 0.14	0.96 ± 0.14	1.06 ± 0.16	1.06 ± 0.16	0.82 ± 0.16	0.81 ± 0.17	112.5 ± 29.3	113.7 ± 27.8
Lampertico 2020 [17]	2.9(0.9)	2.9(0.9)	226(92)	211(87)	124/244(51)	143/241(59)	120(49)	125(51)	0.87(0.72–0.99)	0.86(0.76-1.00)		
Byun 2022 [18]	18(20.7)	8(9.2)	74(85.1)	149(85.6)	60(69.0)	126(72.4)	52(59.8)	105(60.3)	0.9(0.8-1.0)	0.9(0.8-1.0)	89 ± 25	93 ± 27

### Quality assessment and publication bias

The Cochrane method of evaluating RCTs indicated that of the four RCTs included in the meta-analysis, 2 studies were deemed at high risk of bias [7, 18]. One study [7] had reporting bias as some of the outcomes mentioned in the trial protocol were not reported in the original paper. In contrast, the other study [18] had selection, performance, and detection biases due to its open-label nature (Fig. S1). Judgments for risk of bias are provided in Table S3.

### Primary outcome

#### Effect on HBV DNA

**Fig. 2:**
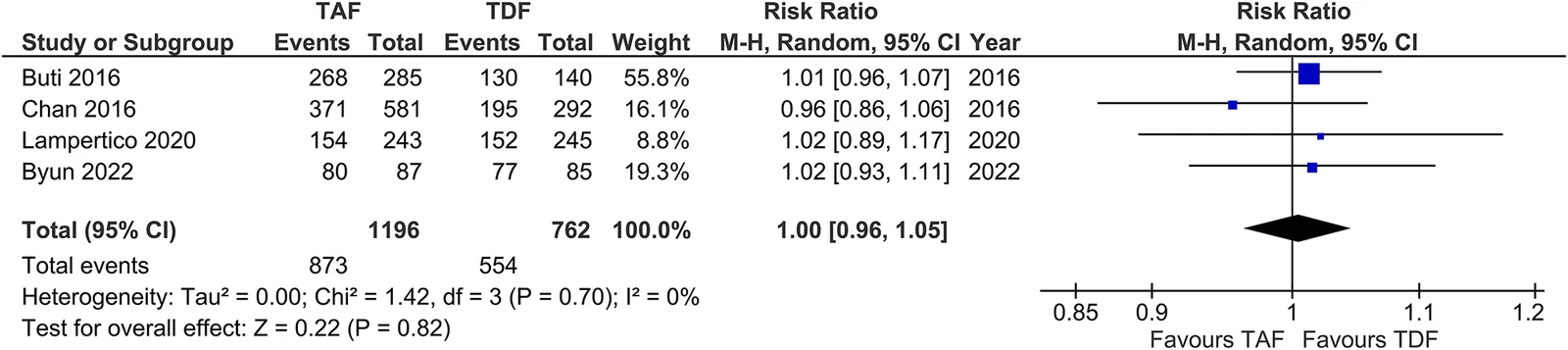
Effect of TAF compared to TDF on virological suppression

All the studies [7, 16–18] reported patients with HBV DNA < 15–29 IU/ml. TAF was non-inferior to TDF regarding virological suppression (RR = 1.00; 95% CI = 0.96 to 1.05; *p* = 0.82; I^2^ = 0%) (Fig. 2).

### Secondary outcomes

#### Effect on ALT normalization

All studies [7, 16–18] reported ALT Normalization. Regarding ALT normalization, TAF seemed more efficacious in normalizing ALT than TDF (RR = 1.38; 95% CI = 1.16 TO 1.64; *p* = 0.0002; I^2^= 18%). (Fig. S2)

#### Effect on Hip BMD

Hip BMD was reported in all the studies [7, 16–18]. TAF showed a significantly smaller reduction in hip BMD than TDF (MD = 1.44; 95%CI = 0.98 to 1.91; *p* < 0.00001; I^2^= 87%). On performing subgroup analysis, TAF showed a significantly smaller decrease in Hip BMD than TDF in patients under 50 years (MD = 1.81; 95% CI = 1.60 to 2.02; *p* < 0.00001; I^2^= 46%). In patients older than 50 years, TAF and TDF showed similar efficacy (MD = 0.84; 95% CI=-0.13 to 1.81; *p* = 0.09; I^2^= 56%). (Fig. S3)

#### Effect on spine BMD

All studies provided data on spine BMD [7, 16–18]. Spine BMD had a significantly smaller decrease with TAF than TDF (MD = 1.93 95% CI = 1.42 to 2.44; *p* < 0.00001; I^2^= 80%). Excluding the Chan 2016 study, heterogeneity became negligible (MD = 1.63; 95% CI = 1.59 to 1.67; *p* < 0.00001; I^2^= 0%). (Fig. S4) The significant decline in heterogeneity after excluding Chan 2016 is due to reporting of younger mean age in this study.

#### Effect on serum creatinine

Serum creatinine was reported in all the studies [7, 16–18]. Serum creatinine levels were found to be significantly less elevated with TAF compared to TDF (MD= -0.02; 95% CI= -0.03 to -0.01; *p* < 0.00001; I^2^= 0%). (Fig. S5)

#### Effect on eGFR

All studies [7, 16, 18] except Lampertico et al. [17] provided data on eGFR. TAF significantly caused a smaller eGFR reduction than TDF (MD = 3.55; 95% CI = 2.97 to 4.14; *p* < 0.00001; I^2^ = 0%). (Fig. S6)

#### Effect on LDL

Except for the study conducted by Byun et al. [18], all other studies [7, 16, 17] reported data from patients whose LDL cholesterol levels were > 190–300 mg/dl (> 190 mg/dl in Lampertico et al. study and > 300 mg/dl in Buti et al. and Chan et al. study). Buti 2016 reported 14/277 events in the TAF arm versus 1/135 in the TDF arm; Chan 2016 reported 23/560 versus 0/282; and Lampertico 2020 reported 9/243 versus 4/245. Pooled across the three trials, this corresponds to 46/1080 events in the TAF group versus 5/662 in the TDF group. The TAF group had a significantly greater proportion of patients with LDL cholesterol levels > 190–300 mg/dl than the TDF group (RR = 4.95; 95% CI = 1.21 to 20.29; *p* = 0.03; I^2^ = 41%). (Fig. S7) The estimate is uncertain due to a few events and wide confidence intervals.

A GRADE summary tool of findings is presented in Table S4.

## Discussion

Our meta-analysis of four RCTs shows that TAF and TDF suppress HBV equally, but TAF has better bone and kidney safety, except for higher LDL levels. Owing to its long-term use, TDF, effective in reducing viral load in both CHB and HIV infection, has been linked to decreased renal function and bone complications [24, 25]. In contrast, TAF has better plasma stability than TDF and can efficiently deliver the active metabolite TFV to the target cells at much lower doses (25 mg) than TDF (300 mg). TAF is non-inferior to TDF in terms of efficacy, thereby reducing the DNA viral load with a better renal and bone safety profile [7, 16].

Our comprehensive review and meta-analysis used data from four RCTs that included 1,960 CHB patients treated with either TDF or TAF. This study aimed to compare the efficacy and safety of TAF versus TDF in CHB. The primary outcome of our study was the proportion of patients with CHB achieving DNA levels below 15–29 IU/ml, and the secondary outcomes of interest were the proportion of patients who achieved ALT normalization, Hip and Spine BMD scores, change in serum creatinine concentration, and change in eGFR. TAF showed significantly improved efficacy and safety for all outcomes except for virological suppression, which demonstrated comparable efficacy to TDF, and for LDL levels, it showed significantly reduced safety compared to TDF.

Both TDF and TAF were able to suppress viral replication and reduce HBV DNA viral load to < 15–29 IU/ml. However, neither treatment group had a significant difference in efficacy. One paper presents a detailed evaluation of two major, phase 3, double-blind RCTs of TAF against TDF in CHB patients, with pooled results demonstrating non-inferiority of TAF in terms of virological suppression compared to TDF [26], which is similar to our pooled result. This effect of virological suppression is achieved via competitive inhibition of HBV DNA polymerase by both drugs [10].

Analysis of all the included studies showed that TAF therapy resulted in a significantly higher proportion of patients achieving ALT normalization than TDF. Saputra et al.‘s systematic review [27] found superior efficacy of TAF compared to TDF in achieving ALT normalization, aligning with our study. Kaneko et al. recently examined the efficacy and safety of switching from TDF to TAF therapy. In this trial, patients in the TAF group had comparable efficacy to TDF in terms of ALT normalization, which may be due to the very small size of this trial to generate meaningful results. The therapeutic significance is that TAF reduces viral load by inhibiting DNA synthesis and hence stopping replication, decreasing inflammation and the risk of hepatic events, making it a potential drug for improving liver function. It is also likely attributable to its intracellular activation mechanism, which concentrates TFV-DP directly within hepatocytes, achieving greater suppression of HBV replication at the site of liver injury [28].

Our study showed that TAF had significantly less reduction in Hip BMD scores as compared to TDF. A subgroup analysis in our study showed that TAF had significant improvement compared to TDF in hip BMD among patients aged less than 50 years, while both classes of drugs showed similar safety profiles in participants over the age of 50 years. Heterogeneity was non-significant and moderately significant in the above subgroups. It should be noted that the included studies differed in design and patient characteristics also. The studies by Buti et al. and Chan et al. evaluated treatment-naïve patients, whereas Lampertico et al. and Byun et al. included patients who had achieved virological suppression on TDF and were subsequently switched to TAF. Therefore, the reduction in heterogeneity observed in the age-based subgroup analysis may not be attributable solely to age differences, as study design and prior TDF exposure may also have contributed to the observed variability in hip BMD outcomes. Similarly, our study found a smaller reduction in spine BMD in the TAF group compared to the TDF group. Heterogeneity was reduced to 0 after excluding the study by Chan et al. [16]. Sensitivity analysis demonstrated that exclusion of the Chan et al. study reduced heterogeneity from 80% to 0% while maintaining the direction and significance of effect (MD = 1.63; 95% CI = 1.59 to 1.67; *p* < 0.00001). Although Chan et al. was judged to be at low risk of bias, its inclusion introduced substantial statistical heterogeneity, suggesting that differences in study characteristics or patient populations rather than methodological quality may have contributed to variability in spine BMD outcomes. The possible reason for this heterogeneity could be due to the study’s large sample size, comprising a younger population with a mean age of 38 years as compared to the other three studies used in the analysis. A study by Fong et al. showed significant improvement in both hip and spine BMD from baseline to week 12 after switching from TDF to TAF and sustained improvement until week 24 [29]. A pooled analysis of 2 studies by Buti et al. found that the TAF group experienced less loss in hip and spine BMD than the TDF group [26]. Similarly, previous studies showed that treatment with TDF had an adverse effect on hip and spine BMD compared to TAF [24, 25, 30]. The mechanism behind this reduction in BMD is that both TAF and TDF reduce the N-terminal propeptide of type 1 collagen, a bone production marker. TAF also reduces the bone resorption marker C-telopeptide, indicating a reduced decrease in BMD [31]. Also, TFV is known for its mitochondrial toxicity and accumulation in renal proximal tubular cells due to active transport processes. This accumulation can damage the cells, reducing phosphate levels and increasing bone turnover, thereby reducing BMD [32–34].

Both TAF and TDF cause a rise in serum creatinine levels. Pooled results of our study showed that patients treated with TAF had significantly less increase in serum creatinine levels than TDF. The difference was non-significant in the study by Buti et al. [7] and Byun et al. [18], while this difference was significant in the study by Chan et al. [16] and Lampertico et al. [17]. The eGFR was reported by 3 studies, and the pooled results of all the studies showed a significantly lesser decline in eGFR in the TAF group vs. TDF group. Liu et al.’s [35] network meta-analysis showed similar results as our meta-analysis in terms of eGFR, while the results are different in the case of serum creatinine, where Liu et al.’s [35] study showed non-significant difference regarding the change in serum creatinine in both TAF and TDF groups. The discrepancy in serum creatinine outcomes could stem from the inclusion of extension trials in the Liu et al. study [35], which accumulates long-term safety data, potentially yielding similar results for both study drugs. This highlights how follow-up duration affects renal outcomes. A possible mechanism behind the effect of these drugs on kidney function is that it leads to a greater increase in serum TFV-DP levels. In contrast, TAF is more stable in serum and directly delivers the metabolite to target cells. Hence, this reduces the concentrations of TFV-DP exposed to kidney tubular cells, thereby reducing kidney damage [32–34].

The lipid-lowering effect of TDF is well-established in the studies done in HIV patients [36, 37]. Our study found significantly beneficial effects in terms of LDL cholesterol in the TDF group compared to the TAF group. A meta-analysis by Hwang et al. [38] showed a significant elevation in LDL cholesterol in the TAF group compared to other NAs. The study by Buti et al. [26] reported increased lipid parameters by the TAF treatment. TDF lowers cholesterol levels by lowering the activity of the CD36/PPAR-alpha pathway, while this effect is not observed in treatment with TAF [39].

There are several key strengths associated with this meta-analysis. The pooling data from the four RCTs in our meta-analysis raised the statistical power of our study while also improving the accuracy of effect size estimation. Our study exhibited low heterogeneity, which indicates that the findings across the included studies were relatively consistent and that the results can be more confidently combined and compared. Also, all the included studies assessed efficacy at 48 weeks, ensuring high consistency and reinforcing our conclusion’s reliability. Several systematic reviews and meta-analyses have previously examined the comparative efficacy and safety of TAF versus TDF in CHB. The pooled analysis by Buti et al. [26] drew from two pivotal phase 3 double-blind RCTs and established non-inferiority of TAF for virological suppression alongside superior bone and renal safety. Saputra et al. [27] similarly demonstrated superior ALT normalization with TAF in a systematic review. More recent network meta-analyses, such as those by Liu et al. [35], have extended comparisons beyond the TAF–TDF dyad, incorporating both RCT and observational data, while others such as Hwang et al. [38] focused exclusively on lipid parameters. However, these prior reviews were characterized by important methodological heterogeneity: several pooled RCT evidence alongside real-world observational data, included patients treated with multiple agents simultaneously, or focused on select outcomes rather than providing a simultaneous evaluation of virological, hepatic, renal, bone, and lipid endpoints within a single RCT-exclusive framework. Crucially, no prior RCT-restricted meta-analysis conducted a formal age-stratified subgroup analysis of bone mineral density outcomes, an important clinical gap given that the impact of antiviral-related bone loss differs substantially across age groups. The present study addresses this gap by: (1) restricting inclusion exclusively to RCTs to minimize confounding inherent in observational designs; (2) simultaneously evaluating all major efficacy and safety domains, HBV DNA suppression, ALT normalization, hip and spine BMD, serum creatinine, eGFR, and LDL cholesterol, within a unified analysis; and (3) conducting an age-stratified (below vs. above 50 years) subgroup analysis of hip BMD outcomes, which, to our knowledge, has not been previously reported in this literature. By maintaining a high-quality, RCT-exclusive evidence base and conducting a prespecified age-stratified analysis of bone outcomes, which revealed that TAF’s bone-protective advantage was statistically significant only in patients under 50 years, this study provides clinically actionable and methodologically rigorous evidence that complements, rather than simply replicates, the conclusions of prior reviews.

There are also several inherent limitations to this study. Due to the limited data available, it could not include other outcomes used to assess bone and kidney function. Moreover, insufficient studies were available for performing meta-analysis on other lipid parameters such as total cholesterol, high-density lipoprotein (HDL) cholesterol, and triglyceride. Therefore, the analysis of lipid parameters as a safety indicator for TAF could only be performed for LDL cholesterol. Our meta-analysis has a relatively small number of studies, which may limit the robustness and generalizability of the findings. Also, Two out of the four studies had high risk of bias, thereby compromising the internal validity of the findings. The search strategy was limited to databases available to the investigators, as Embase, Scopus, and Web of Science were inaccessible. Due to less available databases, the researcher could only get four relevant RCTs. Hence, no funnel was drawn. Another important limitation is the clinical heterogeneity among the included studies. Two studies (Buti and Chan) evaluated treatment-naïve patients with high baseline HBV DNA levels, whereas the studies by Lampertico and Byun included patients who had already achieved viral suppression on TDF and were subsequently switched to TAF. Although this difference appeared to have minimal impact on virological outcomes, likely due to the high antiviral efficacy of both agents, it may have substantially influenced bone-related outcomes. Patients in the switch studies had prior exposure to TDF, which is associated with reductions in bone mineral density, and therefore any observed improvements following the switch to TAF may partly reflect recovery from previous TDF-related bone effects rather than the intrinsic superiority of TAF. This clinical heterogeneity may have contributed to the high heterogeneity observed in hip and spine BMD analyses. All included trials had a follow-up duration of only 48 weeks, insufficient to capture long-term consequences such as fracture incidence, renal function decline, or cardiovascular events. Moreover Most patients were young and healthy at baseline, not representative of real-world patients with cirrhosis or pre-existing kidney disease. Lastly, all four trials were sponsored by the manufacturer of both study drugs, raising the possibility of sponsorship-related bias in trial design, outcome selection, or reporting, patient reported outcomes (adherence, quality of life) despite their randomized, controlled design.

## Conclusion

In conclusion, our findings indicate that in patients with CHB, TAF provides similar antiviral efficacy to TDF, with better renal and bone safety, but may adversely affect lipid levels. Further large and long-term studies are needed. While TAF was associated with significantly smaller reductions in hip and spine BMD and a more favorable renal trajectory compared with TDF, these findings should be interpreted with appropriate caution, as BMD percentage change, serum creatinine, and eGFR are surrogate markers rather than hard clinical endpoints. None of the included trials were powered or designed to capture fragility fractures, progression to chronic kidney disease, or dialysis initiation, and the 48-week follow-up duration is too short to establish whether these biochemical and densitometric differences translate into meaningfully reduced long-term skeletal or renal morbidity. The clinical relevance of TAF’s safety advantages therefore remains to be confirmed through trials incorporating hard outcomes and extended observation periods. The higher proportion of patients with elevated LDL cholesterol in the TAF group should similarly be considered within the broader framework of cardiovascular risk rather than as an isolated laboratory abnormality. LDL elevation is a recognized surrogate for atherosclerotic risk, but none of the included trials reported adjudicated cardiovascular events, validated risk scores, or long-term lipid trajectories, making it difficult to determine whether this signal translates into measurable cardiovascular morbidity. To fully assess TAF’s long-term efficacy and safety, further research is warranted, particularly in special populations, such as individuals with compromised liver, kidney, and bone function, as well as pregnant women. These insights underscore the need for ongoing investigation to optimize treatment strategies for CHB patients.

## Supplementary Information

Below is the link to the electronic supplementary material.

Supplementary Material 1

Supplementary Material 2

## Data Availability

All data generated or analysed during this study are included in this published article and its supplementary information files.

## References

[1] Jefferies M, Rauff B, Rashid H, Lam T, Rafiq S. Update on global epidemiology of viral hepatitis and preventive strategies. World J Clin cases. 2018;6(13):589. 10.12998/wjcc.v6.i13.589 PMC6232563 30430114

[2] Hepatitis B, September | WHO. Accessed 7th 2023 am. https://www.who.int/news-room/factsheets/detail.

[3] Kwon SY, Lee CH. Epidemiology and prevention of hepatitis B virus infection. Korean J Hepatol. 2011;17(2):87. 10.3350/kjhep.2011.17.2.87 PMC3304633 21757978

[4] Nelson NP, Easterbrook PJ, McMahon BJ. Epidemiology of hepatitis B virus infection and impact of vaccination on disease. Clin Liver Dis. 2016;20(4):607–28. 10.1016/j.cld.2016.06.006 PMC5582972 27742003

[5] Shiffman ML. Approach to the patient with chronic hepatitis B and decompensated cirrhosis. Liver Int. 2020;40:22–6. 10.1111/liv.14359 32077612

[6] Wilkins T, Sams R, Carpenter M. Hepatitis B: screening, prevention, diagnosis, and treatment. Am Family Phys. 2019;99(5):314–23. 30811163

[7] Buti M, Gane E, Seto WK, Chan HL, Chuang WL, Stepanova T, Hui AJ, Lim YS, Mehta R, Janssen HL, Acharya SK. Tenofovir alafenamide versus tenofovir disoproxil fumarate for the treatment of patients with HBeAg-negative chronic hepatitis B virus infection: a randomised, double-blind, phase 3, non-inferiority trial. Lancet Gastroenterol Hepatol. 2016;1(3):196–206. 10.1016/S2468-1253(16)30107-8 28404092

[8] Duraisamy GS, Bhosale D, Lipenská I, Huvarova I, Růžek D, Windisch MP, Miller AD. Advanced therapeutics, vaccinations, and precision medicine in the treatment and management of chronic hepatitis b viral infections; where are we and where are we going? Viruses. 2020;12(9):998. 10.3390/v12090998 PMC7552065 32906840

[9] Nguyen MH, Wong G, Gane E, Kao JH, Dusheiko G. Hepatitis B virus: advances in prevention, diagnosis, and therapy. Clin Microbiol Rev. 2020;33(2):10–128. 10.1128/CMR.00046-19 PMC7048015 32102898

[10] Delaney IVWE, Ray AS, Yang H, Qi X, Xiong S, Zhu Y, Miller MD. Intracellular metabolism and in vitro activity of tenofovir against hepatitis B virus. Antimicrob Agents Chemother. 2006;50(7):2471–7. 10.1128/AAC.00138-06 PMC1489769 16801428

[11] Fung S, Kwan P, Fabri M, Horban A, Pelemis M, Hann HW, Gurel S, Caruntu FA, Flaherty JF, Massetto B, Kim K. Tenofovir disoproxil fumarate (TDF) vs. emtricitabine (FTC)/TDF in lamivudine resistant hepatitis B: A 5-year randomised study. J Hepatol. 2017;66(1):11–8. 10.1016/j.jhep.2016.08.008 27545497

[12] Sax PE, Zolopa A, Brar I, Elion R, Ortiz R, Post F, Wang H, Callebaut C, Martin H, Fordyce MW, McCallister S. Tenofovir alafenamide vs. tenofovir disoproxil fumarate in single tablet regimens for initial HIV-1 therapy: a randomized phase 2 study. JAIDS J Acquir Immune Defic Syndr. 2014;67(1):52–8. 10.1097/QAI.0000000000000225 24872136

[13] Chan HLY, Buti M, Lim YS, Agarwal K, Marcellin P, Brunetto M, Chuang WL, Janssen HLA, Fung S, Izumi N, Abdurakhmanov D. Long-term treatment with tenofovir alafenamide for chronic hepatitis B results in high rates of viral suppression and favorable renal and bone safety. Am J Gastroenterol. 2024;119(3):486–96. 10.14309/ajg.0000000000002468 PMC10903997 37561058

[14] Buti M, Riveiro-Barciela M, Esteban R. Tenofovir alafenamide fumarate: a new tenofovir prodrug for the treatment of chronic hepatitis B infection. J Infect Dis. 2017;216(suppl8):S792–6. 10.1093/infdis/jix135 29156043

[15] Murakami E, Wang T, Park Y, Hao J, Lepist EI, Babusis D, Ray AS. Implications of efficient hepatic delivery by tenofovir alafenamide (GS-7340) for hepatitis B virus therapy. Antimicrob Agents Chemother. 2015;59(6):3563–9. 10.1128/AAC.00128-15 PMC4432166 25870059

[16] Chan HL, Fung S, Seto WK, Chuang WL, Chen CY, Kim HJ, Hui AJ, Janssen HL, Chowdhury A, Tsang TY, Mehta R. Tenofovir alafenamide versus tenofovir disoproxil fumarate for the treatment of HBeAg-positive chronic hepatitis B virus infection: a randomised, double-blind, phase 3, non-inferiority trial. lancet Gastroenterol Hepatol. 2016;1(3):185–95. 10.1016/S2468-1253(16)30024-3 28404091

[17] Lampertico P, Buti M, Fung S, Ahn SH, Chuang WL, Tak WY, Ramji A, Chen CY, Tam E, Bae H, Ma X. Switching from tenofovir disoproxil fumarate to tenofovir alafenamide in virologically suppressed patients with chronic hepatitis B: a randomised, double-blind, phase 3, multicentre non-inferiority study. lancet Gastroenterol Hepatol. 2020;5(5):441–53. 10.1016/S2468-1253(19)30421-2 32087795

[18] Byun KS, Choi J, Kim JH, Lee YS, Lee HC, Kim YJ, Yoo BC, Kwon SY, Gwak GY, Lim YS. Tenofovir alafenamide for drug-resistant hepatitis B: a randomized trial for switching from tenofovir disoproxil fumarate. Clin Gastroenterol Hepatol. 2022;20(2):427–37. 10.1016/j.cgh.2021.04.045 33962041

[19] Terrault NA, Bzowej NH, Chang KM, Hwang JP, Jonas MM, Murad MH. AASLD guidelines for treatment of chronic hepatitis B. Hepatology (Baltimore MD). 2016;63(1):261. 10.1002/hep.28156 PMC5987259 26566064

[20] European Association For The Study Of The Liver. EASL clinical practice guidelines: management of chronic hepatitis B virus infection. J Hepatol. 2012;57(1):167–85. 10.1016/j.jhep.2012.02.010 22436845

[21] Liaw YF, Kao JH, Piratvisuth T, Chan HL, Chien RN, Liu CJ, Gane E, Locarnini S, Lim SG, Han KH, Amarapurkar D. Asian-Pacific consensus statement on the management of chronic hepatitis B: a 2012 update. Hep Intl. 2012;6:531–61. 10.1007/s12072-012-9365-4 26201469

[22] Lampertico P, Agarwal K, Berg T, Buti M, Janssen HL, Papatheodoridis G, Zoulim F, Tacke F. EASL 2017 Clinical Practice Guidelines on the management of hepatitis B virus infection. J Hepatol. 2017;67(2):370–98. 10.1016/j.jhep.2017.03.021 28427875

[23] Terrault NA, Lok AS, McMahon BJ, Chang KM, Hwang JP, Jonas MM, Brown RS Jr, Bzowej NH, Wong JB. Update on prevention, diagnosis, and treatment of chronic hepatitis B: AASLD 2018 hepatitis B guidance. Hepatology. 2018;67(4):1560–99. 10.1002/hep.29800 PMC5975958 29405329

[24] Marcellin P, Gane EJ, Flisiak R, Trinh HN, Petersen J, Gurel S, Kaita KD, Kotzev IA, Tsai N, Flaherty JF, Schall RE. Long Term Treatment with Tenofovir Disoproxil Fumarate for Chronic Hepatitis B Infection is Safe and Well Tolerated and Associated with Durable Virologic Response with no Detectable Resistance: 8 Year Results from Two Phase 3 Trials: 229. Hepatology. 2014;60:A313–4.

[25] Fung S, Kwan P, Fabri M, Horban A, Pelemis M, Hann HW, Gurel S, Caruntu FA, Flaherty JF, Massetto B, Dinh P. Randomized comparison of tenofovir disoproxil fumarate vs emtricitabine and tenofovir disoproxil fumarate in patients with lamivudine-resistant chronic hepatitis B. Gastroenterology. 2014;146(4):980–8. 10.1053/j.gastro.2013.12.028 24368224

[26] Buti M, Lim YS, Chan HLY, Agarwal K, Marcellin P, Brunetto MR, Chuang WL, Janssen HLA, Fung SK, Izumi N, Jablkowski MS. Eight-year efficacy and safety of tenofovir alafenamide for treatment of chronic hepatitis B virus infection: Final results from two randomised phase 3 trials. Aliment Pharmacol Ther. 2024;60(11–12):1573–86. 10.1111/apt.18278 PMC11599788 39327857

[27] Saputra RC, Hidayat N. Evaluation Effect of Tenofovir Alafenamide (TAF) in Long-Term Therapy for Chronic Hepatitis B: A Systematic Review. Indonesian J Gastroenterol Hepatol Dig Endoscopy. 2022;23(3):216–25.

[28] Kaneko S, Kurosaki M, Tamaki N, Itakura J, Hayashi T, Kirino S, Osawa L, Watakabe K, Okada M, Wang W, Shimizu T. Tenofovir alafenamide for hepatitis B virus infection including switching therapy from tenofovir disoproxil fumarate. J Gastroenterol Hepatol. 2019;34(11):2004–10. 10.1111/jgh.14686 31017689

[29] Fong TL, Lee BT, Tien A, Chang M, Lim C, Ahn A, Bae HS. Improvement of bone mineral density and markers of proximal renal tubular function in chronic hepatitis B patients switched from tenofovir disoproxil fumarate to tenofovir alafenamide. J Viral Hepatitis. 2019;26(5):561–7. 10.1111/jvh.13053 30576085

[30] Sax PE, Wohl D, Yin MT, Post F, DeJesus E, Saag M, Pozniak A, Thompson M, Podzamczer D, Molina JM, Oka S. Tenofovir alafenamide versus tenofovir disoproxil fumarate, coformulated with elvitegravir, cobicistat, and emtricitabine, for initial treatment of HIV-1 infection: two randomised, double-blind, phase 3, non-inferiority trials. Lancet. 2015;385(9987):2606–15. 10.1016/S0140-6736(15)60616-X 25890673

[31] Soldado-Folgado J, Rins-Lozano O, Arrieta-Aldea I, Gonzále-Mena A, Cañas-Ruano E, Knobel H, Garcia-Giralt N, Güerri-Fernández R. Changes in bone quality after switching from a TDF to a TAF based ART: A pilot randomized study. Front Endocrinol. 2023;14:1076739. 10.3389/fendo.2023.1076739 PMC10083312 37051195

[32] Hall AM, Hendry BM, Nitsch D, Connolly JO. Tenofovir-associated kidney toxicity in HIV-infected patients: a review of the evidence. Am J Kidney Dis. 2011;57(5):773–80. 10.1053/j.ajkd.2011.01.022 21435764

[33] Fernandez-Fernandez B, Montoya-Ferrer A, Sanz AB, Sanchez-Nino MD, Izquierdo MC, Poveda J, Sainz-Prestel V, Ortiz-Martin N, Parra-Rodriguez A, Selgas R, Ruiz-Ortega M. Tenofovir nephrotoxicity: 2011 update. AIDS research and treatment. 2011;2011. 10.1155/2011/354908 PMC3119412 21716719

[34] Haskelberg H, Hoy JF, Amin J, Ebeling PR, Emery S, Carr A, STEAL Study Group. Changes in bone turnover and bone loss in HIV-infected patients changing treatment to tenofovir-emtricitabine or abacavir-lamivudine. PLoS ONE. 2012;7(6):e38377. 10.1371/journal.pone.0038377 PMC3376146 22719882

[35] Liu Z, Zhao Z, Ma X, Liu S, Xin Y. Renal and bone side effects of long-term use of entecavir, tenofovir disoproxil fumarate, and tenofovir alafenamide fumarate in patients with hepatitis B: a network meta-analysis. BMC Gastroenterol. 2023;23(1):384. 10.1186/s12876-023-03027-4 PMC10638829 37950196

[36] Santos JR, Saumoy M, Curran A, Bravo I, Llibre JM, Navarro J, Estany C, Podzamczer D, Ribera E, Negredo E, Clotet B. The lipid-lowering effect of tenofovir/emtricitabine: a randomized, crossover, double-blind, placebo-controlled trial. Clin Infect Dis. 2015;61(3):403–8. 10.1093/cid/civ296 25870325

[37] Tungsiripat M, Kitch D, Glesby MJ, Gupta SK, Mellors JW, Moran L, Jones L, Alston-Smith B, Rooney JF, Aberg JA. A pilot study to determine the impact on dyslipidemia of adding tenofovir to stable background antiretroviral therapy: ACTG 5206. AIDS. 2010;24(11):1781. 10.1097/QAD.0b013e32833ad8b4 PMC2913151 20495438

[38] Hwang EG, Jung E, Yoo JJ, Kim SG, Kim YS. Risk of dyslipidemia in chronic hepatitis B patients taking tenofovir alafenamide: a systematic review and meta-analysis. Hep Intl. 2023 Apr;26:1–0. 10.1007/s12072-023-10528-7 37099248

[39] Shaheen AA, AlMattooq M, Yazdanfar S, Burak KW, Swain MG, Congly SE, Borman MA, Lee SS, Myers RP, Coffin CS. Tenofovir disoproxil fumarate significantly decreases serum lipoprotein levels compared with entecavir nucleos (t) ide analogue therapy in chronic hepatitis B carriers. Aliment Pharmacol Ther. 2017;46(6):599–604. 10.1111/apt.14218 28707319

