# Supplementary material for: Efficacy and safety of tenofovir alafenamide versus tenofovir disoproxil in chronic hepatitis b: a systematic review and meta-analysis of randomized controlled trials
Source: BMC Infect Dis. 2026 Jul 6;26:1734. doi: 10.1186/s12879-026-13974-4 (PMC13621647; doi:10.1186/s12879-026-13974-4)
Supplement: Supplementary file 1 [file 12879_2026_13974_MOESM1_ESM.docx]

Supplementary Table S1: Detailed search strategy

| Database | Search Strategy | Results |
| --- | --- | --- |
| PubMed | (((("Hepatitis B"[MeSH Terms] OR "Hepatitis B virus"[MeSH Terms] OR (hepatitis b) OR (Hepatitis B Virus)) OR (HBV))) | 11659 |
|  | OR ((Hepatitis B) AND (Tenofovir Alfenamide)) OR |  |
|  | (Tenofovir alafenamide fumarate) OR (TAF)) AND |  |
|  | (Tenofovir disoproxil)) OR ((Tenofovir Disoproxil |  |
|  | Fumarate)) OR (TDF) |  |
| Google Scholar | "hepatitis b"\|"Hepatitis B Virus"\|"HBV"\|"Hepatitis B" "Tenofovir Alfenamide"\|"Tenofovir alafenamide fumarate"\|"TAF" "Tenofovir disoproxil"\|"Tenofovir Disoproxil Fumarate"\|"TDF" | 5150 |
| Cochrane | "hepatitis B":ti,ab,kw OR "hepatitis B virus":ti,ab,kw | 11315 |
|  | AND tenofovir alafenamide fumarate:ti,ab,kw AND |  |
|  | "tenofovir disoproxil fumarate":ti,ab,kw OR "tenofovir |  |
|  | disoproxil":ti,ab,kw (Word variations have been |  |
|  | searched) |  |

Supplementary Table S2: Characteristics of RCTs

| Characteristics | Buti  2016 | Chan  2016 | Lampertico  2020 | Byun  2022 |  |
| --- | --- | --- | --- | --- | --- |
|  |  |  |  |  |  |
| Study name | Tenofovir alafenamide versus tenofovir disoproxil fumarate for the treatment of patients with HBeAg-negative chronic hepatitis B virus infection: a randomized, double-blind, phase 3, non-inferiority trial. | Tenofovir alafenamide versus tenofovir disoproxil fumarate for the treatment of HBeAg-positive chronic hepatitis B virus infection: a randomized, double-blind, phase 3, non-inferiority trial. | Switching from tenofovir disoproxil fumarate to tenofovir alafenamide in virologically suppressed patients with chronic hepatitis B: a randomised, double-blind, phase 3, multicentre non-inferiority study | Tenofovir Alafenamide for Drug-Resistant Hepatitis B: A Randomized Trial for Switching From Tenofovir Disoproxil Fumarate |  |
| Patients, n | 425 | 873 | 488 | 174 |  |
| Enrollment initiation | 2013 | 2013 | 2016 | 2018 |  |
| Enrollment completion | 2014 | 2014 | 2017 | 2018 |  |
| Year of completion | 2022 | 2022 | 2020 | 2021 |  |
| Population | Patients aged at least 18 years with HBeAg-negative chronic HBV infection. | Patients aged 18 years and above with chronic HBV infection positive for the hepatitis B antigen (HBeAg). | Patients were at least 18 years old with documented evidence of HBeAg-negative or HBeAg- positive chronic HBV infection including those with compensated cirrhosis | All patients had a positive serum HBsAg test for at least 6 months. Patients were required to be between 20 to 80 years old with well preserved liver and renal function |  |
| Trial type | Randomized, double-blind, non-inferiority, multi-center study | Randomized, double-blind, non-inferiority, multi-center study | Randomized, double-blind, non-inferiority, multi-center study | Randomized, double-blind, non-inferiority, multi-center study |  |
| Inclusion  criteria | Patients were aged at least 18 years with HBeAg negative chronic HBV infection (with plasma HBV DNA concentrations of >20000 IU/mL), serum ALT concentrations of greater than 60 U/L in men or greater than 38 U/L in women and at no more than ten times the upper limit of normal [ULN], and estimated creatinine clearance of at least 50 mL/min (by the Cockcroft-Gault method). Treatment-naive (less than 12 weeks of previous therapy) as well as treatment- experienced patients (those who previously received treatment with any oral antiviral agent for HBV infection for ≥12 weeks). Previous interferon use was allowed if stopped at least 6 months before study baseline  Assessments were eligible between September 12th, 2013, and October 31st, 2014. | Patients aged at least 18 years (with no upper age limit) with HBeAg-positive chronic hepatitis B infection (with HBV DNA concentrations of at least 20 000 IU/mL), alanine aminotransferase (ALT) concentrations of higher than 60 U/L in men or higher than 38 U/L in women and at most ten times the upper limit of normal (ULN), and estimated creatinine clearance of at least 50 mL/min (by the Cockcroft-Gault method). Patients who had not previously received treatment for HBV infection and those who had been previously treated or were undergoing treatment for HBV infection were eligible between September 11th, 2013, to Dec 20th, 2014. | Patients were at least 18 years of age with documented evidence of HBeAg-negative or HBeAg-positive chronic HBV infection, including those with compensated cirrhosis. Eligible patients had been receiving tenofovir disoproxil fumarate 300 mg once a day for at least 48 weeks, and as monotherapy for at least 24 weeks before screening. Eligible patients were virally suppressed (HBV DNA <lower limit of quantification [LLOQ] by local laboratory assessment) for at least 12 weeks before screening and were required to have HBV DNA <20 IU/mL (by central laboratory) at screening. Eligible patients had an estimated creatinine clearance of at least 50 mL/min (by the Cockcroft-Gault method). | All patients had history of documented ETV resistance mutations (rtT184A/C/F/G/I/L/S, rtS202G, or rtM250L/V, on the presence of rtM204V/I) or ADV resistance mutations (rtA181V/T and/or rtN236T) before preceding TDF therapy. All patients had a positive serum HBsAg test for at least 6 months. Patients were required to be between 20 and 80 years old with well preserved liver function (Child-Pugh class A) and renal function (estimated creatinine clearance by Cockcroft Gault formula 50 mL/min). |  |
| Exclusion criteria | Patients with platelet counts of 50000 cells per μL or less, hemoglobin less than 10 g/dL, albumin less than 3 g/dL, direct bilirubin of greater than 2·5 times the ULN, and aspartate aminotransferase (AST) or ALT greater than ten times the ULN. Patients with evidence of decompensation (ie, clinical ascites, encephalopathy or variceal hemorrhage) and those with hepatocellular carcinoma as well as patients co-infected with hepatitis C, hepatitis D, or HIV. | Patients with platelet counts of 50 000 cells per μL or less, hemoglobin of less than 10 g/dL, albumin of less than 3 g/dL, and total bilirubin of more than 2·5 times the ULN. Patients with evidence of decompensation (ie, clinical ascites, encephalopathyor variceal hemorrhage) and those with hepatocellular carcinoma | Patients with hepatocellular carcinoma, evidence of hepatic decompensation, or co-infection with other viruses (HIV, hepatitis C virus [HCV], or hepatitis D virus [HDV]) were excluded, as were patients with abnormal laboratory parameters, including haemoglobin of less than 10 g/dL, absolute neutrophil count of less than 750 cells per mm³, platelet count of 50 000 per mm³ or less, aspartate aminotransferase (AST) or serum alanine aminotransferase (ALT) greater than 5 × the upper limit of normal (ULN), albumin less than 3·0 mg/dL, International Normalised Ratio (INR) greater than 1·5 × ULN (unless stable on anticoagulant regimen), or total bilirubin >2·5 × ULN). | Patients were excluded if they met any of the following criteria: (1) coinfection with hepatitis C virus, hepatitis D virus, or human immunodeficiency virus (HIV); (2) decompensated liver disease; (3) treatment with immunosuppressive drugs; (4) previous diagnosis of hepatocellular carcinoma (HCC); or (5) solid organ or bone marrow transplantation. |  |
| Treatment | Patients received tenofovir alafenamide 25 mg orally once daily or tenofovir disoproxil fumarate 300 mg orally once daily | 25 mg tenofovir alafenamide or 300 mg tenofovir disoproxil fumarate orally once daily. All patients received placebo tablets matching the alternative treatment (ie, patients assigned to receive tenofovir alafenamide also received a matching tenofovir disoproxil fumarate placebo tablet, and vice versa). | Patients received either tenofovir alafenamide 25 mg plus matching tenofovir disoproxil fumarate placebo or tenofovir disoproxil fumarate 300 mg plus matching tenofovir alafenamide matching placebo orally once daily. | Patients were randomly assigned in a 1:1 ratio to either switching to TAF 25 mg once daily (TAF group) or continuing TDF 300 mg once daily (TDF group) through interactive web response system using a centralized procedure |  |
| Primary outcome | Proportion of patients with HBV DNA less than 29 IU/mL at week 48 of treatment, as determined by COBAS Taqman HBV test for use with the High Pure System (Roche Molecular Systems, Inc; Pleasanton, CA, USA) with a lower limit of quantitation of 29 IU/mL and a lower limit of detection of 10 IU/mL. | Proportion of patients with HBV DNA less than 29 IU/mL at week 48 of treatment as determined by PCR. The lower limit of quantitation of the assay was 29 IU/mL, and the lower limit of detection was 10 IU/mL. | Proportion of patients who lost virological control (ie, HBV DNA ≥20 IU/mL) at week 48, as defined by the modified US Food and Drug Administration (FDA)-defined snapshot algorithm | Proportion of patients who maintained serum HBV DNA <60 IU/mL at week 48 as determined by local laboratory and AASLD 2018 criteria |  |
| Secondary outcome | Percentage change in hip bone mineral density, percentage change in spine bone mineral density, and change from baseline in serum creatinine. Proportion of patients with ALT normalization at week 48, proportion of patients with plasma HBV DNA less than 29 IU/mL (target not detected), the proportion of patients with HBsAg loss, the proportion of patients with HBsAg seroconversion to anti-HBs. | Proportion of patients with HBeAg loss and with HBeAg seroconversion to anti-HBe at week 48. Percent change in spine bone mineral density, and change from baseline in serum creatinine. Proportion of patients with plasma HBV DNA less than 29 IU/mL (target not detected), the proportion of patients with HBsAg  seroconversion to anti-HBs at week 48, the change from baseline in fibrosis the incidence of drug-resistant mutations in patients who had HBV DNA of 69 IU/mL or higher at week 48, and the proportion of patients with ALT normalization at week 48. | Proportion of patients with HBV DNA less than 20 IU/mL (including the proportion with target not detected), by missing equals failure analysis, proportion of patients with HBeAg loss and seroconversion to anti-HBe, proportion of patients with HBsAg loss and sero-conversion to anti-HBs, patients with normal ALT and normalised ALT and change from baseline in fibrosis. The percentage change from baseline in hip and spine bone mineral density and change in creatinine clearance at week 48. | Proportion of patients with undetectable serum HBV DNA (<15 IU/mL), incidence of virologic breakthrough, changes from baseline in ALT levels, the proportion of patients with normal ALT levels, the patients with HBeAg seroclearance/seroconversion in basal HBeAg-positive patients, the patients with HBsAg seroclearance and changes in hip and spine BMD, eGFR, body weight, and lipid profiles from baseline at week 48. Virologic breakthrough was defined as an increase in serum HBV DNA levels - 1 log10 IU/mL from nadir during continued treatment. |  |
| Intervention Period | 8 years with efficacy endpoints assessed through 48 weeks in the trial | 8 years with efficacy endpoints assessed through 48 weeks in the trial | 8 years with efficacy endpoints assessed through 48 weeks in the trial | 5 years with efficacy endpoints assessed through 48 weeks in the trial |  |

**Supplementary Table S3: Risk of Bias Judgements for Included RCTs**

| **Domain** | **Chan 2016** | **Buti 2016** | **Lampertico 2020** | **Byun 2022** |
| --- | --- | --- | --- | --- |
| **Random sequence generation** | Low — computer-generated randomization reported | Low — Computer-generated allocation sequence | Low — computer-generated randomization reported | Low — Interactive web response system using a centralized  procedure |
| **Allocation concealment** | Low — Automated treatment was  assignment via an interactive voice web response system | Low — Automated treatment was  assignment via an interactive voice web response system | Low — Automated treatment was  assignment via an interactive voice web response system | Low — Interactive web response system using a centralized  procedure |
| **Blinding of participants & personnel** | Low — double-blind; matching placebo was used | Low — double-blind; matching placebo was used | Low — double-blind; matching placebo was used | **High** — open-label design |
| **Blinding of outcome assessment** | Low — Outcomes were lab values (objective assessment) and blinding was done | Low — Outcomes were lab values (objective assessment) and blinding was done | Unclear — Outcomes were lab values (objective assessment), however nothing mentioned about blinding | **High** — open-label |
| **Incomplete outcome data** | Low — Trial profile mentions the drop outs with reasons and explanation | Low — Trial profile mentions the drop outs with reasons and explanation | Low — Trial profile mentions the drop outs with reasons and explanation | Low — Trial profile mentions the drop outs with reasons and explanation |
| **Selective reporting** | Low — All outcomes mentioned are reported as well | **High** — HBsAg and fibrotest not reported after the analysis whereas mentioned as ouctomes | Low — All outcomes mentioned are reported as well | Low — All outcomes mentioned are reported as well |
| **Other bias** | Low — baseline characteristics equal proportion with no conflicts of Interest | Low — baseline characteristics equal proportion with no conflicts of Interest | Low — baseline characteristics equal proportion with no conflicts of Interest | Low — baseline characteristics equal proportion with no conflicts of Interest |
| **Overall judgement** | Low Risk | **High Risk** | Low Risk | **High risk** |

**Supplementary Table S4: GRADE Summary of Findings**

| **Outcome (contributing studies)** | **Effect estimate (95% CI)** | **Risk of bias** | **Inconsistency** | **Indirectness** | **Imprecision** | **Certainty (GRADE)** |
| --- | --- | --- | --- | --- | --- | --- |
| **HBV DNA suppression (Chan, Buti, Lampertico, Byun)** | RR 1.00 (0.96–1.05) | Seriousᵃ | Not serious (I²=0%) | Not serious | Not serious | ⊕⊕⊕◯ Moderate |
| **ALT normalization (Chan, Buti, Lampertico, Byun)** | RR 1.38 (1.16–1.64) | Seriousᵃ | Not serious (I²=18%) | Not serious | Not serious | ⊕⊕⊕◯ Moderate |
| **Hip BMD (Chan, Buti, Lampertico, Byun)** | MD 1.44 (0.98–1.91) | Seriousᵃ | Seriousᵇ (I²=87%) | Not serious | Not serious | ⊕⊕◯◯ Low |
| **Spine BMD (Chan, Buti, Lampertico, Byun)** | MD 1.93 (1.42–2.44) | Seriousᵃ | Seriousᵇ (I²=80%) | Not serious | Not serious | ⊕⊕◯◯ Low |
| **Serum creatinine (Chan, Buti, Lampertico, Byun)** | MD −0.02 (−0.03 to −0.01) | Seriousᵃ | Not serious (I²=0%) | Not serious | Not serious | ⊕⊕⊕◯ Moderate |
| **eGFR (Chan, Buti, Byun)** | MD 3.55 (2.97–4.14) | Seriousᶜ | Not serious (I²=0%) | Not serious | Not serious | ⊕⊕⊕◯ Moderate |
| **LDL cholesterol >190–300 mg/dL (Chan, Buti, Lampertico)** | RR 4.95 (1.21–20.29) | Not seriousᵅ | Seriousᵇ (I²=41%) | Not serious | Very seriousᵉ | ⊕◯◯◯ Very low |

ᵃ Downgraded for risk of bias because Byun 2022 contributes to this outcome and was rated high risk for blinding of participants/personnel and blinding of outcome assessment (open-label design).

ᵇ Downgraded for substantial unexplained heterogeneity; for spine BMD, sensitivity analysis excluding Chan 2016 reduced I² from 80% to 0% without changing the direction or significance of effect.

ᶜ Downgraded for risk of bias because two of the three contributing trials (Buti 2016, Byun 2022) were rated high risk overall, including high risk of detection bias in Byun 2022.

ᵅ Not downgraded for risk of bias: the trial rated high risk among the three contributors (Buti 2016) was judged high risk due to selective reporting of unrelated outcomes (HBsAg, FibroTest), not due to bias in LDL measurement itself; for the domain most relevant to this objective lab outcome (blinding of outcome assessment), the three contributing trials were rated Low, Low, and Unclear — none High.

ᵉ Downgraded two levels for very serious imprecision: only 51 total LDL events across 1,742 patients (46/1,080 TAF vs 5/662 TDF), producing a wide confidence interval (1.21–20.29) compatible with effects ranging from minimal to very large.
