# Supplementary material for: Efficacy and safety of tenofovir alafenamide versus tenofovir disoproxil in chronic hepatitis b: a systematic review and meta-analysis of randomized controlled trials
Source: BMC Infect Dis. 2026 Jul 6;26:1734. doi: 10.1186/s12879-026-13974-4 (PMC13621647; doi:10.1186/s12879-026-13974-4)
Supplement: Supplementary file 2 [file 12879_2026_13974_MOESM2_ESM.docx]

***Figure S1.*** Risk of Bias Graph and Summary

***Figure S2.*** Effect of TAF compared to TDF on ALT Normalization

***Figure S3.*** Subgroup analysis of the effect of TAF compared to TDF on Hip BMD

***Figure S4A.*** Effect of TAF compared to TDF on spine BMD

***Figure S4B.*** Sensitivity analysis of the effect of TAF compared to TDF on spine BMD.

***Figure S5.*** Effect of TAF compared to TDF on serum creatinine.

***Figure S6.*** Effect of TAF compared to TDF on eGFR

***Figure S7.*** Effect of TAF compared to TDF on LDL cholesterol.
